# Reviews and Book Notices

**Published:** 1880-04

**Authors:** 


### Reviews and Book Notices.

ARTICLE XIII.—PHOTOGRAPHIC ILLUSTRATIONS OF SKIN DISEASES. By GEO. HENRY FOX, A.M., M.D., Clinical Professor of Dermatology, Starling Medical College, Columbus, Ohio, etc., etc. *Part* 5. Eczema Infantile; E. Papulosum; E. Ichorosum; E. Pustulosum, and E. Squamosum. *Part* 6. Eczema Barbæ; E. Manum; E. e. Venis Varicosis; Ulcus Varicosum; Psoriasis Annulata.

The lately published parts of this admirable atlas of colored skin portraits, are fully equal in merit to those which first were placed in the hands of the profession, and which gave promise of so much in the future. We have heretofore had occasion to notice the latter with an expression of our approval and pleasure. If any one who happens to inspect them, feels inclined to take exception to the contrast between the symptoms exhibited by the art of the photographer and those portrayed by the skill of the lithographer, he should remember that, after all, the photograph exactly represents the phenomena, color excepted, and that the classical symptoms displayed in several of the chromolithographs are, as a matter of fact, rarely seen in the consultation-room of the practitioner or in the clinic of the college or dispensary.

In the portrait of eczema of the palm, the character and extent of the skin infiltration is admirably shown; but one of the best of this series is the portrait of eczema of the beard, which is so truthful that one with little experience in these matters, could almost make a differential diagnosis between the disease represented and that produced by either a vegetable parasite or constitutional syphilis.

We congratulate the author, and his co-laborer in the matter of coloring, Dr. J. Gaertner, upon the success which they have attained not only on paper, but in the large demand for the work which the publishers have been sufficiently fortunate to satisfy.

